# Observation of multiple bulk bound states in the continuum modes in a photonic crystal cavity

**DOI:** 10.3762/bjnano.14.45

**Published:** 2023-04-27

**Authors:** Rui Chen, Yi Zheng, Xingyu Huang, Qiaoling Lin, Chaochao Ye, Meng Xiong, Martijn Wubs, Yungui Ma, Minhao Pu, Sanshui Xiao

**Affiliations:** 1 Department of Electrical and Photonics Engineering, Technical University of Denmark, DK-2800 Kgs. Lyngby, Denmark; 2 State Key Lab of Modern Optical Instrumentation, Centre for Optical and Electromagnetic Research, College of Optical Science and Engineering, International Research Center for Advanced Photonics, Zhejiang University, Hangzhou 310058, China; 3 NanoPhoton – Center for Nanophotonics, Technical University of Denmark, Kgs. Lyngby, Denmark

**Keywords:** bound states in the continuum, bulk modes analysis, photonic crystal

## Abstract

Obtaining bound states in the continuum (BICs) in photonic crystals gives rise to the realization of resonances with high quality factors for lasing and nonlinear applications. For BIC cavities in finite-size photonic crystals, the bulk resonance band turns into discrete modes with different mode profiles and radiation patterns. Here, photonic-crystal BIC cavities encircled by the photonic bandgap of lateral heterostructures are designed. The mirror-like photonic bandgap exhibits strong side leakage suppression to confine the mode profile in the designed cavity. Multiple bulk quantized modes are observed both in simulation and experiment. After exciting the BIC cavity at different positions, different resonance peaks are observed. The physical origin of the dependence between the resonance peak and the illuminating position is explained by analyzing the mode profile distribution and further verified by numerical simulations. Our findings have potential applications regarding the mode selectivity in BIC devices to manipulate the lasing mode in photonic-crystal surface-emitting lasers or the radiation pattern in nonlinear optics.

## Introduction

Optical bound states in the continuum (BICs) are localized waves whose energy is embedded in a radiation region and which are completely decoupled from radiating waves [1]. They could be regarded as discrete states of Fano resonances evolved to be completely orthogonal to the continuum [2]. These modes have infinite quality factors (Q factors) and cannot be excited from the external radiative continuum, thereby providing efficient ways to trap light and to enhance light–matter interactions [3–7]. Multiple wave systems, such as water wave [8], phononic [9], acoustic [10], and photonic [3–4] systems, can support BICs, which play a critical role in sensors [11–13], filters [14], lasers [15–17], nonlinear optics [18–24], and quantum devices [25–26]. An ideal BIC could be viewed as a cavity that suppresses radiation in all directions. For planar BIC devices, suitable topological constellations can ensure low radiation in the vertical direction, while infinite periods ensure vanishing transverse leakage, leading theoretically to an infinite Q factor. However, realistic devices inevitably have only a finite size, which introduces lateral leakage at the outgoing boundary. Consequently, BIC modes with infinite Q factor turn into quasi-BICs that are accessible from the radiative continuum [27–28]. In addition to the degradation of the Q factor, the finite-size effect also turns the continuous photonic bands of the infinite device into discrete energy levels, similar to the quantization of electronic states in quantum dots. Each quantized BIC mode has its specific bulk mode profile and radiation pattern. Combining a photonic bandgap perimeter with the finite-size BIC cavity could significantly prevent transverse leakage, thus giving rise to ultrahigh Q factors [29–30].

Photonic crystals (PhCs) are composed of periodic unit cells that modulate the propagation of electromagnetic waves by defining allowed and forbidden energy bands. Because of the slow group velocity, photons are confined in the transverse direction at the band edge of the PhC. By carefully designing the resonant properties of the unit cell, a vanishing coupling of resonant modes in each unit cell to all out-of-plane radiation channels can be achieved, forming a BIC mode [31–32]. Such a vanishing coupling could result from symmetry incompatibility between the eigenmode profile of the unit cell and the outgoing radiation [18,23,33] or from the destructive interference of two or more leaky waves that cancel each other in the far field [15–16,34]. PhC-based BIC devices have recently attracted great attention since they can achieve both large-*Q* resonances and strong light confinement in a relatively simple way. Such strong resonances endow PhC-based BIC devices with a strong enhancement of light–matter interaction, indicating great potential for applications in ultrasensitive molecular fingerprint detection [12–13,35], hyperspectral biosensing imaging [36], novel flat light-emitting devices [15–17,37], and nonlinear light generation [18,22–23,38–40].

In this paper, we propose a design of symmetry-protected BIC cavities consisting of aluminum gallium arsenide (AlGaAs) nanoblocks on a sapphire substrate. Motivated by the idea of in-plane leakage suppression by a reflective boundary around the BIC cavity [29–30], a bandgap mirror and transition area surrounding the designed BIC cavity are proposed. We numerically evaluate the bulk band diagram of the corresponding infinite BIC structure as well as the multiple quantized bulk mode profiles of the finite-size BIC cavity. To further verify the multiple modes in the designed cavity, a series of samples is fabricated and tested. Multiple resonances could be observed by translating the excitation spot on the device. Finally, the physical origin of the dependence between the spectral resonance and the illumination position is investigated.

## Results

### Unit cell

**Figure 1:**
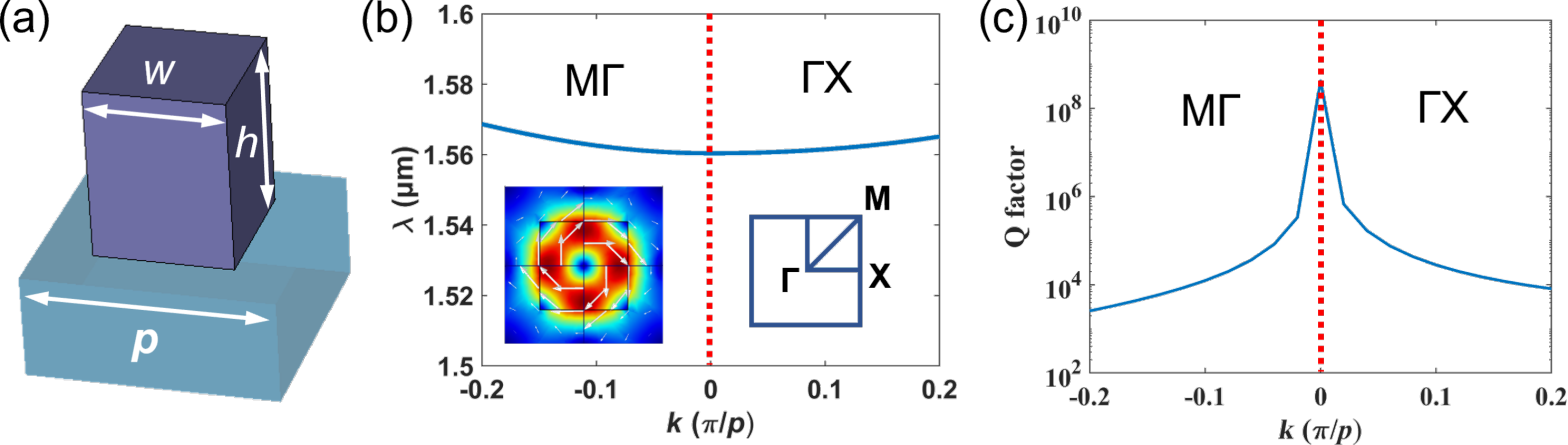
Design of the unit cell. (a) Schematic of a unit cell consisting of an AlGaAs nanoblock on a sapphire substrate. The side length and the height of the nanoblock are *w* and *h*, respectively. The period of the unit is *p*. (b) Band diagram related to the MD BIC mode. The electric field distribution for the mode at the Г point is shown in the inset, where the white arrows indicate the in-plane *E* field vectors. (c) *Q*_r_ of the BIC mode as a function of *k*.

We design a symmetry-protected BIC mode supported by AlGaAs nanoblocks in a square lattice, as schematically depicted in Figure 1a. The side length and the height of the nanoblocks are *w* = 400 nm and *h* = 500 nm, respectively. The lattice constant is *p* = 720 nm. Such a periodic nanostructure array supports a BIC around 1.56 μm and has an infinite Q factor at the Г point, as shown in Figure 1b and Figure 1c, respectively. The electric field (*E* field) distribution of the eigenmode is shown in the inset of Figure 1b, and the white arrows indicate the in-plane *E* field vectors. The annular *E* field distribution of the mode corresponds to a magnetic dipole (MD) with its dipole moment along the vertical direction. The symmetry nature of the MD is incompatible with the illumination waves. Hence, a BIC mode is formed at the Г point. By changing the in-plane *k* vector of the incident field away from the Г point, a small extent of symmetry breaking occurs and the Q factor decreases, as shown in Figure 1c. The Q factor discussed above is the radiative Q factor (*Q*_r_), which only depends on the ideal mode radiation loss. The Q factor of any realistic device is influenced by both the radiative part *Q*_r_ and a nonradiative part *Q*_nr_ via 1/*Q* = 1/*Q*_r_ + 1/*Q*_nr_ provided that the material is lossless. *Q*_nr_ incorporates defects such as structural disorder, surface roughness, and fabrication errors. The simulation of the unit cell was performed by COMSOL Multiphysics with periodic boundary conditions and the eigenfrequency solver.

### Bandgap mirror-assisted finite-size BIC cavity

**Figure 2:**
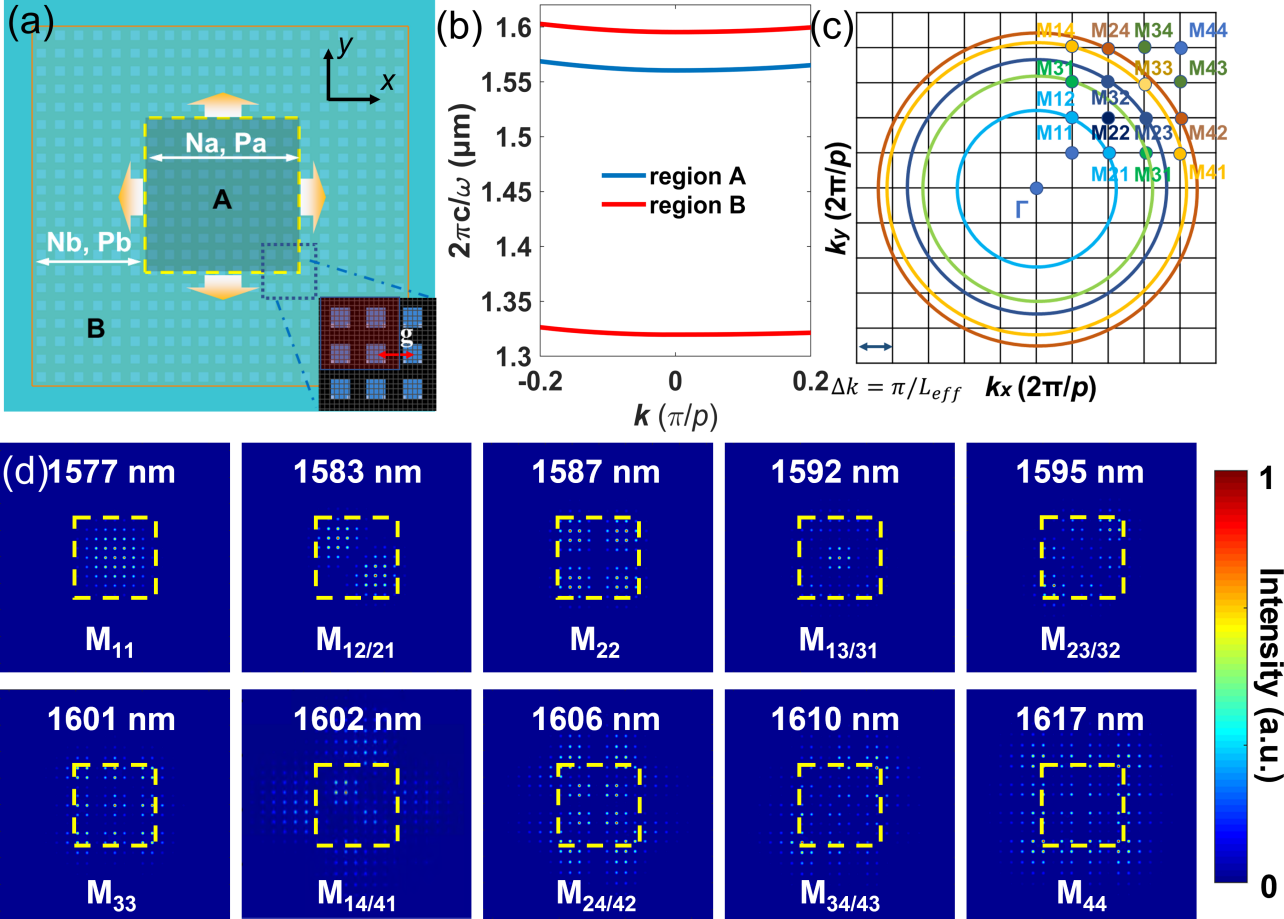
Design of the bandgap mirror-assisted BIC cavity. (a) Schematic of the BIC cavity surrounded by the photonic bandgap mirror. Regions A and B are the BIC cavity and the bandgap heterostructure, respectively. *N*_a_, *N*_b_, *P*_a_ = *p*, and *P*_b_ are the number of nanoblocks and lattice periods in regions A and B, respectively. Inset: the transition region at the interface between the two regions. (b) Band diagram of the structures in regions A and B. (c) The momentum distribution of all bulk modes. (d) The near-field *H* field intensity distribution of modes M_11_ to M_44_. The yellow dashed squares indicate the boundary of region A.

To boost the Q factor of the cavity, a lateral heterostructure with a photonic bandgap, shown as region B in Figure 2a, is introduced to surround the BIC cavity, named region A. The bandgap mirror is composed of the same nanoblocks as illustrated in Figure 1a, but with a different period *P*_b_ = 800 nm, as shown in Figure 2a. Considering the simulation resources, the numbers of nanoblocks in regions A (*N*_a_) and B (*N*_b_) were chosen to be nine and six, respectively. The band diagrams of the structures in regions A and B presented in Figure 2b indicate that the energy of the BIC structures in region A is embedded in the bandgap of region B, thus suppressing in-plane leakage. A transition region with a gap (*g* = 760 nm) between region A and region B is introduced to compensate the momentum mismatch at the heterostructure interface, as drawn in the inset of Figure 2a.

The in-plane momentum of an infinite system could be selected to any value. However, in a finite-lattice system, the continuous band of structures splits into a series of discrete modes located at discrete points with distance δ*k* = π/*L*_eff_ in the momentum space according to the effective size of the system *L*_eff_, as shown in Figure 2c. Each mode could be interpreted as a wave packet composed of the fast oscillating part of the eigenmode of the infinite periodic system |*k*⟩ and a spatially slowly varying envelope function (bulk mode) ϕ(**r**, *k*) [29], which gives:


[1] $$|\psi \left(r\right)\rangle =\sum_{k}\phi \left(r,k\right)|k\rangle ,$$

where k = (*p*π/*L*_eff_, *q*π/*L*_eff_), and *p* and *q* are integers that label the quantized wavevector *k* near the band edge. In our design, the MD supported by the structures is the fast oscillating part |*k*⟩. For the envelop function ϕ, we utilize the indices (*p*, *q*) indicating the corresponding bulk modes localized near (*p*π/*L*_eff_, *q*π/*L*_eff_) in the first quadrant. The locations in momentum space and the magnetic field (*H* field) distributions of several of the lowest-order M_pq_ modes are drawn in Figure 2c and Figure 2d, respectively. The mode indices and eigenwavelengths are marked in each mode pattern. The yellow dashed squares represent region A in the devices. The eigenmodes of the bandgap mirror-assisted finite-size BIC structure are calculated and shown in Figure 2d. This part of the simulation was performed using Ansys Lumerical FDTD. In the simulation, a magnetic dipole cloud with momentum along the *z* direction was applied as the source. Perfectly matched layers are used at all boundaries of the simulation region. The mode patterns are captured by adding a field monitor at the middle plane of the structure. An apodization window is utilized in the monitor to filter out the incident power.

Light blocking by region B could be easily observed for the lower-order modes whose indices are lower than (3,3). For higher-order modes, large mode profiles need a larger size in region B to build a higher potential barrier to confine the light. Due to *C*_4_ symmetry of the structure, all M_pq_ and M_qp_ modes are degenerate, that is, they reside on an iso-frequency contour of the bulk band of the infinite structure, as Figure 2c shows. The mode indices also reveal the bulk mode nodes in the *x* and *y* directions, as shown in Figure 2d. The presented mode profiles of degenerate modes are the superpositions of the mode profiles of the two corresponding modes. As the total mode indices [(p^2^ + q^2^)^1/2^] increase, the mode location in the reciprocal space is further away from the Г point. This leads to a longer eigenwavelength, as seen in the dispersion relationship of the BIC mode in Figure 2b. In our simulation, most of the modes follow this trend except for M_14/41_ and M_33_. As presented in Figure 2d, the large mode pattern of the M_14/41_ mode cannot be contained in the BIC cavity anymore, as the edge of the mode profile penetrates inside the bandgap heterostructure to make the effective size of the mode *L*_eff_ larger. Consequently, the M_33_ mode in the reciprocal space is closer to the Г point than the M_14/41_ modes. For mode indices larger than M_14/41_, mode leakage becomes more significant, resulting in Q factor degradation of the corresponding higher-order bulk modes.

### Sample fabrication and measurement

**Figure 3:**
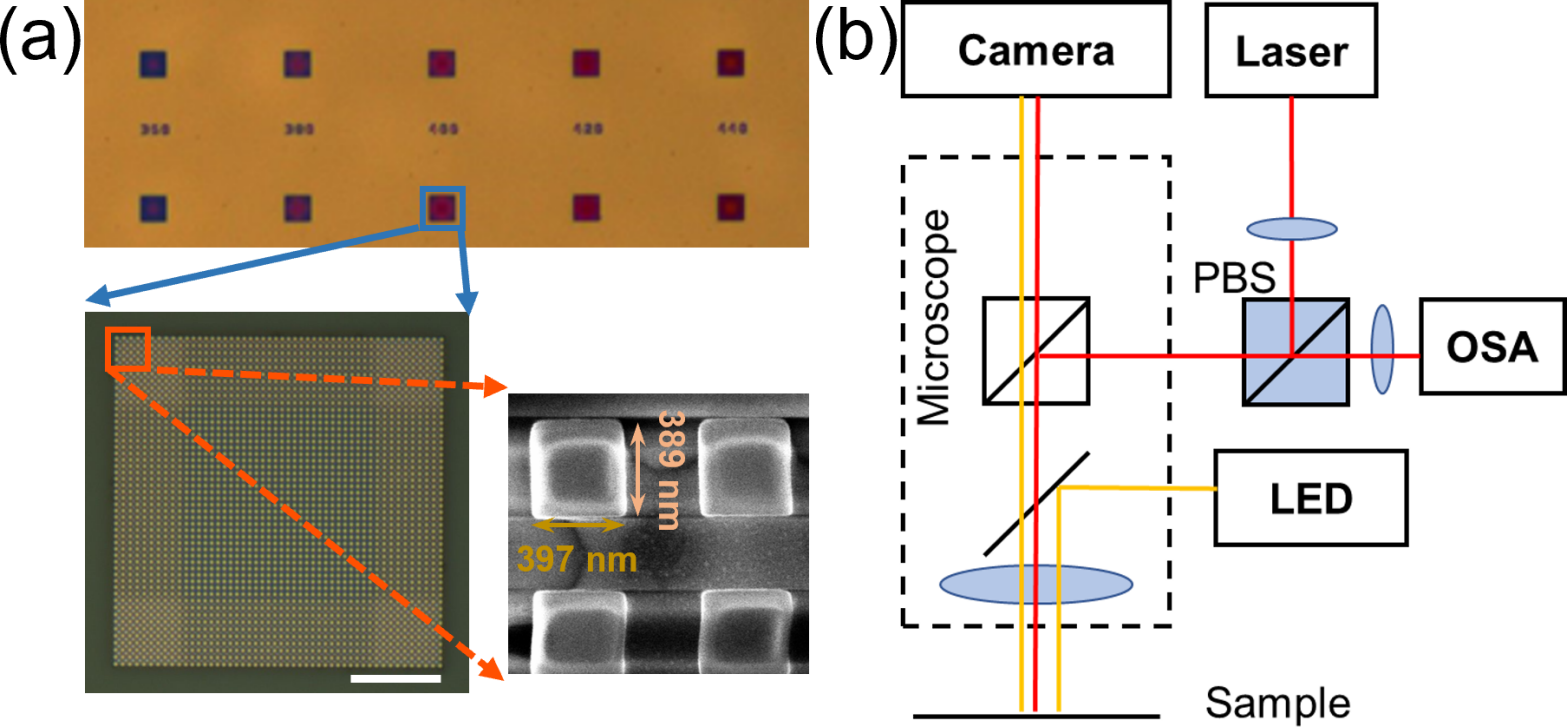
Fabricated samples and optical setup. (a) Optical and scanning electron microscopy images of fabricated samples, scale bar: 10 μm. (b) Diagram of the experimental setup. The yellow and red lines indicate the imaging and measurement light paths, respectively. PBS: polarization-dependent beam splitter; OSA: optical spectrum analyzer.

To verify the multiple modes in the bandgap mirror-assisted BIC cavity, several samples with different sidelengths (*w*) were fabricated by electron beam lithography (EBL) and inductively coupled plasma (ICP) etching on 500 nm thick AlGaAs on a sapphire wafer. The parameter sweep of *w* around 400 nm was carried out to compensate for deviations between fabricated and designed values. To obtain devices with larger Q factors and higher scattering power, cavities with larger *N*_a_ = 30 and *N*_b_ = 10 were fabricated. Optical and scanning electron microscopy (SEM) images are given in Figure 3a. A microscope was refitted to test the scattering spectrum of the sample, as illustrated in Figure 3b. The yellow and red lines represent the imaging and measurement optical paths in the microscope. The imaging light path was utilized to adjust the illuminating position on the sample. In the measurement light path, a polarization-dependent beam splitter (PBS) is added, not only to combine incident and reflection light, but also to filter out the co-polarization component with the incident light. Only the scattering light possessing both polarization components can partially pass the PBS and be detected by the spectrometer. The direct reflective light retaining the same polarization with incidence is reflected by the PBS. Consequently, the interference between the radiation of resonances and direct reflection could be greatly suppressed, and a better signal-to-noise ratio (SNR) and symmetrical Lorentzian resonances could be obtained. A 20× objective was used in the measurement.

**Figure 4:**
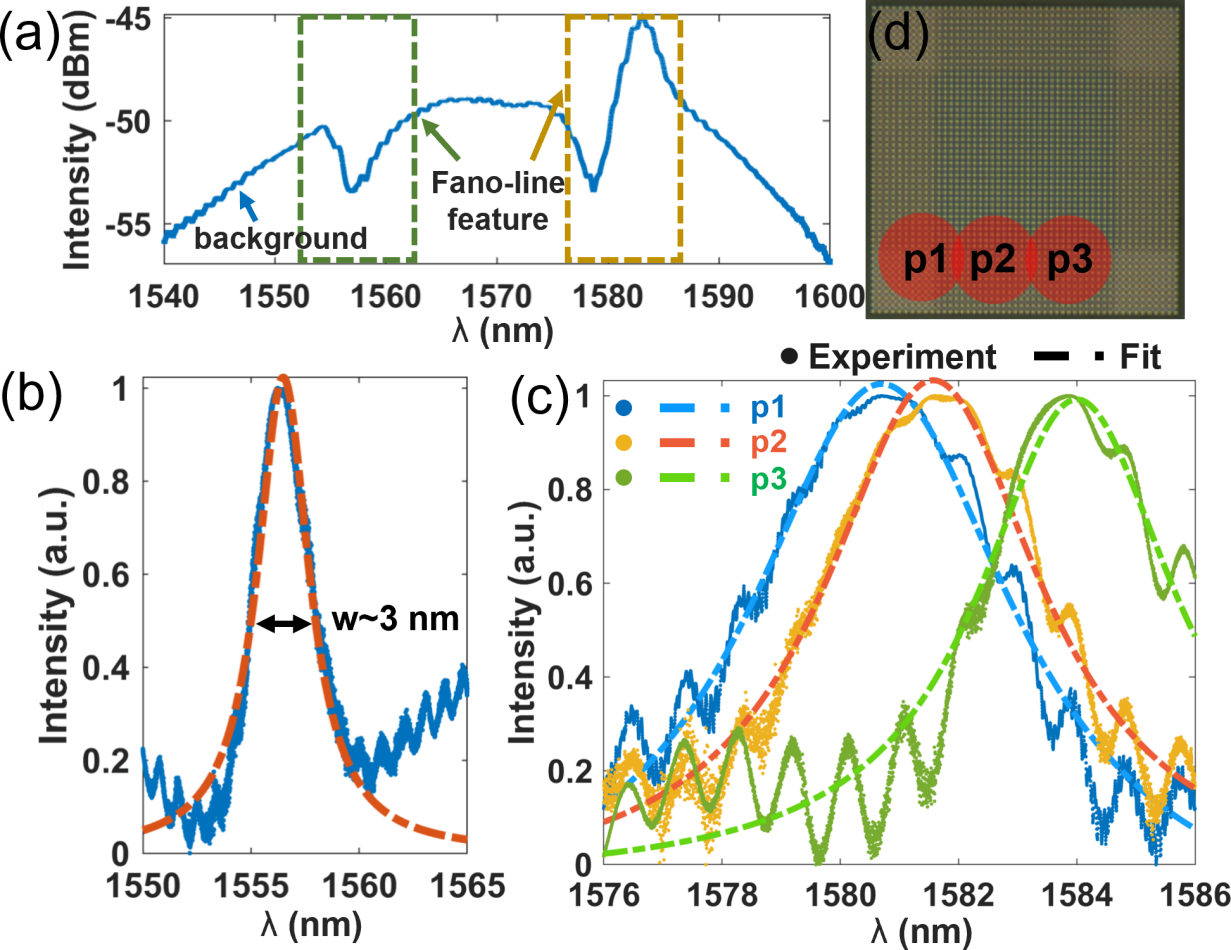
Measurement results. (a) Scattering spectrum over a wide range of wavelengths. (b, c) Resonances observed around 1555 and 1582 nm respectively. Measured data and Lorentzian fittings are shown as dots and dashed lines, respectively. High intensity is used to identify the resonance wavelength in the measurement of (a). (d) Illumination spot positions p1, p2, and p3, which excite the resonances in (c). Fano-line features are observed due to the interference between the designed resonance and direct reflection from the substrate in the measurement of (a). Low intensity and precise illumination position adjustment were applied in the measurements of (b) and (c).

Limited by the excitation efficiencies of one specific position for all modes and the sensitivity of the spectrometer, the weak scattering signal from the resonances is inundated by noise. To identify the resonance wavelength of the cavity, a high-power pulsed laser is applied to obtain stronger signals, thus making the resonance feature more obvious in the spectrum. However, high intensity of direct reflection caused by the pulsed laser could not be totally filtered out with the PBS and, therefore, a pronounced background intensity profile is observed in the measured spectrum shown in Figure 4a. The interference between the background and the scattering of the excited mode leads to the Fano-line features in the spectra. In the measured spectrum, resonances features could be found around 1555 and 1582 nm. To eliminate the background influence and to obtain clear Lorentzian resonances, we applied a low-power broadband light source and finely tuned the illumination spot to obtain maximum excitation and collection efficiency of the setup for each mode. Different from the method applied in [30], the illumination spot size is not the same as the cavity since the large cavity size (ca. 20 μm) would need a large defocus or an objective with low magnification, which seriously degrades the collection efficiency. By finely scanning the illumination position on the sample, resonances were obtained around 1555 and 1582 nm, as presented in Figure 4b and Figure 4c. Interestingly, the resonance peaks around 1582 nm shift in the spectra when the position of the excitation spot is gradually varied, as shown in Figure 4c. The reason of this dependence is the different excitation efficiency at the different illumination positions, which is elaborated below in the Discussion section. The positions of the illumination spots p1, p2, and p3 are schematically marked in Figure 4d. The full width at half maximum (FWHM) of each resonance peak is extracted by numerically fitting the spectrum to a Lorentzian function. The FWHM of the peak in Figure 4b is about 2.9 nm, corresponding to a Q factor of 534. The FWHM of the resonances at 1581, 1582, and 1584 nm in Figure 4c are about 5.5, 4.5, and 4.1 nm, respectively, corresponding to Q factors of 250, 286, and 387. The *Q*_r_ value of each mode could be roughly obtained by extracting the value in Figure 1c at


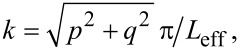


which is larger than 10^4^. ANSYS simulations of the same device could yield more accurate *Q*_r_ values of each mode, but only at computational cost that is too high for normal computers. The lower quality factor of the fabricated samples compared to the simulations in Figure 1c is due to the nonradiative part of the *Q* factor mentioned above. *Q*_nr_ is caused by structural imperfections and disorders, including roughness on the surface, tilted sidewalls, and incomplete etching of the AlGaAs layer, as shown in Figure 3a. Theoretically, resonances of high-order modes have lower Q factors and longer eigenwavelengths than the low-order modes. This trend is well followed by the low-order mode in Figure 4b and the higher-order modes in Figure 4c. However, the resonances in Figure 4c show, counterintuitively, higher Q factors for the resonances at longer wavelength. This might be due to a different influence of fabrication disorders on each mode. The close eigenwavelength values of the resonances in Figure 4c make the *Q*_r_ degradation difference negligible, but the structural disorders (non-symmetry, tilted angle, and center shift of the structure) have a distinct impact on the different modes. If the structural defects are mainly located at the mode node of a specific mode, the loss caused by the defect will decrease. In contrast, disorders at an antinode will lead to obvious loss (small *Q*_nr_) and a dominant Q factor degradation. For our sample, a higher loss caused by defects occurs for the mode with shorter eigenwavelength, yielding higher Q factors to the resonances at longer wavelength. Fabry–Pérot resonances of the substrate (thickness *d* = 600 μm, refractive index *n* = 1.75), with wavelength intervals of (λ^2^/2*nd*) ≈ 1.1 nm lead to the oscillations with a period of about 1 nm in Figure 4.

## Discussion

**Figure 5:**
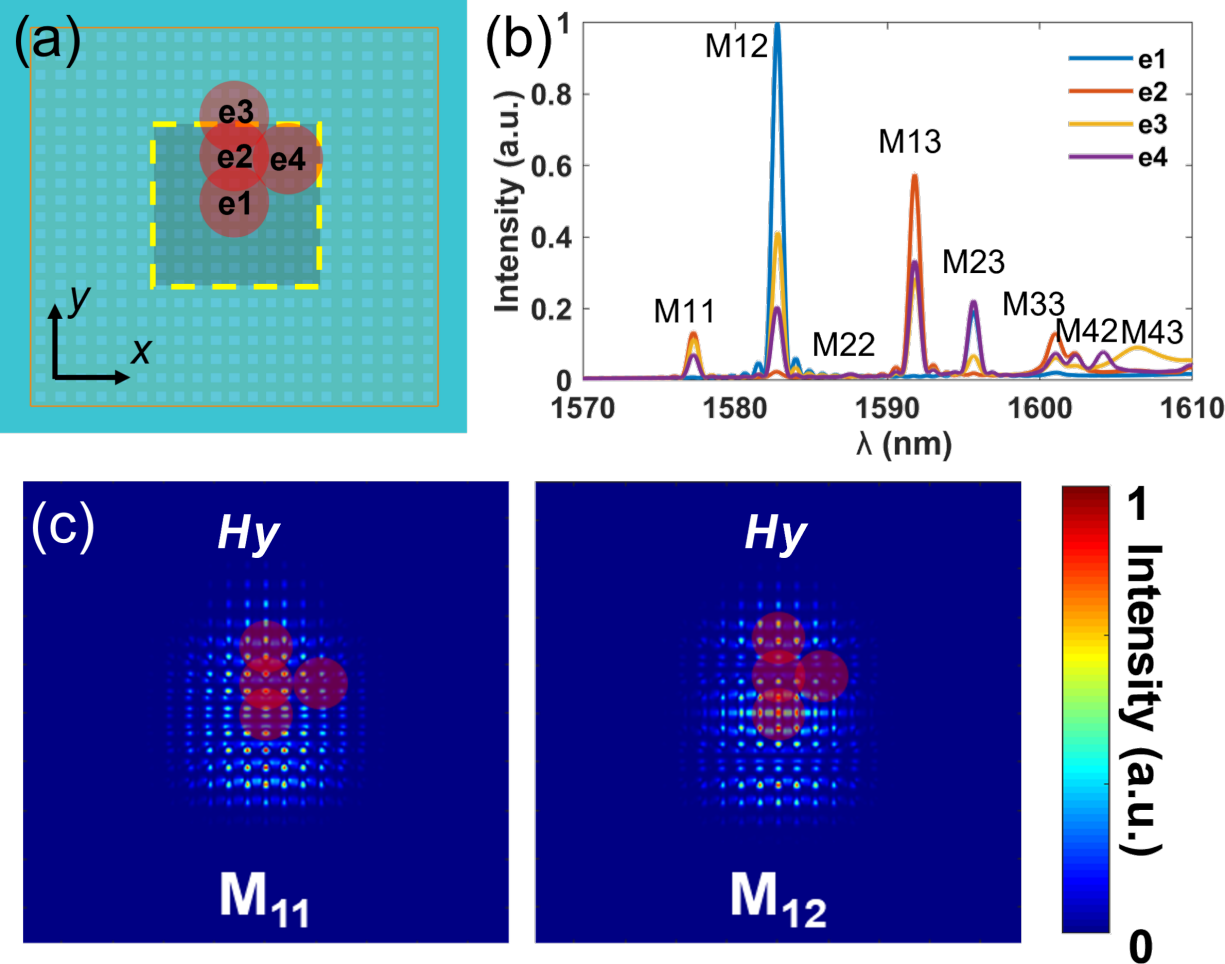
Mode excitation efficiency analysis. (a) Schematic of the illumination positions on the designed cavity. (b) Resonance spectra of the cavity under Gaussian beam excitation with *y*-polarized H-field positions e1 to e4 shown in (a). (c) The *y*-polarized *H* field mode intensity profiles of modes M_11_ and M_12_.

Excitation efficiencies of different modes are responsible for the dependence of the observed resonance wavelength on the excitation position. More overlap between the mode profile and the illumination spot correlates to a higher excitation efficiency of the corresponding mode. As elaborated, the simulation of the designed cavities under Gaussian beam illumination with different positions was carried out. To generally demonstrate this phenomenon, four representative excitation positions, that is, e1(0, 0), e2(0, 2 μm), e3(0, 4 μm), and e4(2 μm, 2 μm), in the eighth symmetry area of the cavity are considered in the simulation, see Figure 5a. The waist radius of the Gaussian beam is 2 μm, which is similar to the focal spot in the experiment. The resonance spectra are extracted in the reflection monitor by applying an apodization window to cut the incident power. It could be observed in these simulations that the mode intensities depend on the position of the optical excitation, as illustrated in Figure 5b. The M_11_ and M_12_ modes are chosen as representatives to explain this dependence. Since the Gaussian beam used in the simulation is *x*-polarized (the *H* field is *y*-polarized), only the *y*-polarized *H* field intensity profiles of M_11_ and M_12_ are given in Figure 5c. The illumination positions e1 to e4 are also marked in Figure 5c. For M_11_, the Gaussian beam at position e1 illuminates the low-intensity area of the M_11_ mode profile of *y* polarization. Hence, the excitation intensity of the M_11_ mode at position e1 is much lower than at the other positions. The same trend could also be observed for the M_12_ mode at the position e2. In contrast, the excitation spot on e1 overlaps the maximum-intensity area of the profile of M_12_, which gives rise to the maximum excitation efficiency for M_12_. The excitation efficiencies of other high-order modes were also qualitatively analyzed. It is noteworthy that theoretically all modes can be excited if the overlap of the mode profile and the illumination spot is not zero. In our measurement, limited by the sensitivity of the setup, only the resonance with the highest excitation efficiency could be observed, while other modes are inundated by noise. The high-order modes have close eigenwavelengths, resulting in the resonance shift in Figure 4c. Theoretically, all modes could be captured in the spectrum, but the excitation and collection efficiency cannot simultaneously reach maximum, thus leading to only four observed modes in the experiment. Applying a transmission setup could decouple the excitation and collection objectives to get maximum excitation and collection efficiency simultaneously, which might be beneficial to capture more modes.

Considering the efficiency of our setup and the computation capacity for simulation, the sizes of the cavity in simulation and experiment are different. However, the dependence of the illumination position and mode excitation efficiency are verified. A computer with higher performance to simulate a larger cavity or improving the efficiency of the setup to test the scattering field of a small cavity could be steps for further studies. Mode excitation efficiency and illumination position dependence enable laser mode modulation in photonic-crystal surface-emitting lasers (PCSELs) [17,41–43], as well as the control of the radiation direction [30] in BIC devices. In our experiment, the mode indices are not identified. They could be identified by observing far-field radiation patterns of the modes [30]. Also, near-field patterns of modes could be obtained by near-field microscopy [44]. Both methods need a precise optical setup and an efficiency improvement of the setup and will be investigated in further studies.

## Conclusion

In this work, multiple quantized modes in PhC-based BIC cavities surrounded by photonic bandgap mirrors are designed and analyzed by a combination of simulations and experiment. Specifically, it is observed in the experiment that the excitation efficiency of the confined modes clearly depends on the position of the illumination spot. To clearly uncover the physical origin of this dependence, Gaussian beams with different positions on the device were simulated, and the overlaps between the excitation and mode profiles with the same polarization were analyzed. The existence of multiple bulk modes in our confined BIC cavities and the strong position dependence of the excitation efficiency of those modes provide an efficient way to control radiation pattern and mode selectivity in the BIC devices.
